# Myocardial Extracellular Volume Estimation by CMR Predicts Functional Recovery Following Acute MI

**DOI:** 10.1016/j.jcmg.2016.06.015

**Published:** 2017-09

**Authors:** Ananth Kidambi, Manish Motwani, Akhlaque Uddin, David P. Ripley, Adam K. McDiarmid, Peter P. Swoboda, David A. Broadbent, Tarique Al Musa, Bara Erhayiem, Joshua Leader, Pierre Croisille, Patrick Clarysse, John P. Greenwood, Sven Plein

**Affiliations:** a Multidisciplinary Cardiovascular Research Centre and Division of Biomedical Imaging, Leeds Institute of Cardiovascular and Metabolic Medicine, University of Leeds, Leeds, United Kingdom; b Department of Medical Physics and Engineering, Leeds Teaching Hospitals NHS Trust, Leeds, United Kingdom; c Université Lyon, INSA Lyon, Université Lyon 1, Université Jean Monnet, CNRS 5220, INSERM 1046, CHU Saint-Etienne, CREATIS, F-69621, Lyon, France

**Keywords:** acute myocardial infarction, cardiovascular imaging agents/techniques, contractile function, CT and MRI, AMI, acute myocardial infarction, AUC, area under the curve, CMR, cardiac magnetic resonance, ECV, extracellular volume, EF, ejection fraction, LGE, late gadolinium enhancement, LV, left ventricle/ventricular, MO, microvascular obstruction, MOLLI, modified Look-Locker inversion

## Abstract

**Objectives:**

In the setting of reperfused acute myocardial infarction (AMI), the authors sought to compare prediction of contractile recovery by infarct extracellular volume (ECV), as measured by T1-mapping cardiac magnetic resonance (CMR), with late gadolinium enhancement (LGE) transmural extent.

**Background:**

The transmural extent of myocardial infarction as assessed by LGE CMR is a strong predictor of functional recovery, but accuracy of the technique may be reduced in AMI. ECV mapping by CMR can provide a continuous measure associated with the severity of tissue damage within infarcted myocardium.

**Methods:**

Thirty-nine patients underwent acute (day 2) and convalescent (3 months) CMR scans following AMI. Cine imaging, tissue tagging, T2-weighted imaging, modified Look-Locker inversion T1 mapping natively and 15 min post–gadolinium-contrast administration, and LGE imaging were performed. The ability of acute infarct ECV and acute transmural extent of LGE to predict convalescent wall motion, ejection fraction (EF), and strain were compared per-segment and per-patient.

**Results:**

Per-segment, acute ECV and LGE transmural extent were associated with convalescent wall motion score (p < 0.01; p < 0.01, respectively). ECV had higher accuracy than LGE extent to predict improved wall motion (area under receiver-operating characteristics curve 0.77 vs. 0.66; p = 0.02). Infarct ECV ≤0.5 had sensitivity 81% and specificity 65% for prediction of improvement in segmental function; LGE transmural extent ≤0.5 had sensitivity 61% and specificity 71%. Per-patient, ECV and LGE correlated with convalescent wall motion score (r = 0.45; p < 0.01; r = 0.41; p = 0.02, respectively) and convalescent EF (p < 0.01; p = 0.04). ECV and LGE extent were not significantly correlated (r = 0.34; p = 0.07). In multivariable linear regression analysis, acute infarct ECV was independently associated with convalescent infarct strain and EF (p = 0.03; p = 0.04), whereas LGE was not (p = 0.29; p = 0.24).

**Conclusions:**

Acute infarct ECV in reperfused AMI can complement LGE assessment as an additional predictor of regional and global LV functional recovery that is independent of transmural extent of infarction.

## Graphical abstract

**Figure:**
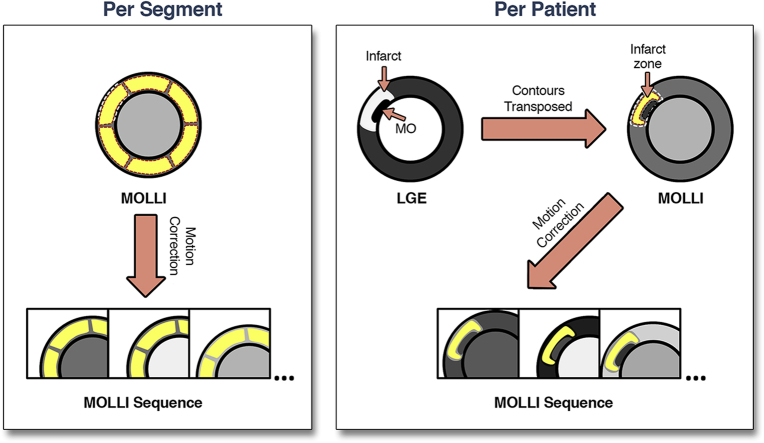

Following reperfused acute myocardial infarction (AMI), the left ventricle (LV) remodels, with the extent of functional recovery in the infarct zone determined by the proportion of viable, contractile cells (1). Late gadolinium enhancement (LGE) cardiac magnetic resonance (CMR) is the clinical reference standard for imaging of myocardial infarction and allows accurate estimation of the infarct extent in vivo (2). The convention of “bright is dead” appears robust in chronic infarction (3), but in AMI, factors such as myocardial edema and the effects of reperfusion therapy add complexity to infarct imaging, reducing the accuracy of LGE to predict recovery of regional wall motion 4, 5, 6. Processes within the infarct zone, such as microvascular obstruction (MO) or intramyocardial hemorrhage, impair functional recovery independent of infarct size 7, 8, demonstrating that differing degrees of infarct “severity” exist. LGE assesses tissue dichotomously as viable or nonviable across the transmural extent of myocardium, but does not consider the severity of tissue damage within the hyperenhanced infarct zone.

Native and post-contrast T1 mapping by CMR allows for estimation of myocardial extracellular volume (ECV). ECV estimation provides the potential for quantitative assessment of severity of tissue disruption and loss of myocytes within the infarct zone, potentially providing an additional dimension of infarct characterization to LGE-derived assessment of transmural extent. ECV mapping has been applied to chronic MI with a range of values in the infarct zone, suggesting that it may be sensitive to severity of tissue damage in myocardial infarction (9), but the method has not been evaluated in patients with AMI.

We hypothesized that ECV estimation by CMR in reperfused ST-segment elevation AMI offers additional predictive value for functional contractile recovery as compared to transmural extent of LGE hyperenhancement.

## Methods

Patients with first ST-segment elevation AMI, revascularized by primary percutaneous coronary intervention within 12 h of onset of pain, were prospectively recruited from a single tertiary center. ST-segment elevation AMI was defined as per current guidelines (10). Exclusion criteria were previous AMI or coronary artery bypass grafting, estimated glomerular filtration rate <30 ml/min/1.73 m^2^, cardiomyopathy, or contraindications to CMR. The study protocol was approved by the institutional research ethics committee and complied with the Declaration of Helsinki; all patients gave written informed consent. Clinical management (including anticoagulation and use of aspiration catheters during primary percutaneous coronary intervention) was at the discretion of the responsible clinician, reflecting contemporary practice and guidelines, and performed blind to CMR results. All patients were considered for beta-blockade, angiotensin-converting enzyme inhibitors, statins, dual antiplatelet therapy, and cardiac rehabilitation. A venous blood sample for hematocrit was obtained at the start of each scan.

### Image acquisition

All patients had CMR at 3.0-T (Achieva TX, Philips Healthcare, Best, the Netherlands) within 3 days of index presentation (acute scan) and the same CMR protocol 3 months post-AMI (convalescent scan). To ensure consistent slice positioning and infarct analysis between time points, modified Look-Locker inversion (MOLLI) T1 mapping, tissue tagging, T2-weighted imaging, and LGE and wall motion cine image acquisition were performed in 3 identical short-axis positions, by acquiring the central 3 slices of 5 parallel short-axis slices spaced equally from mitral annulus to LV apical cap (11). In addition, LGE and cine imaging were performed using a contiguous stack of short-axis slices covering the whole LV. The same slice geometry, position, and 10-mm slice thickness were used for all sequences. Post-contrast T1 mapping was performed 15 min after contrast administration, and LGE imaging at 16 to 20 min. Pulse sequence parameters and imaging protocol are described in the Online Appendix.

### Image analysis

**Figure 1:**
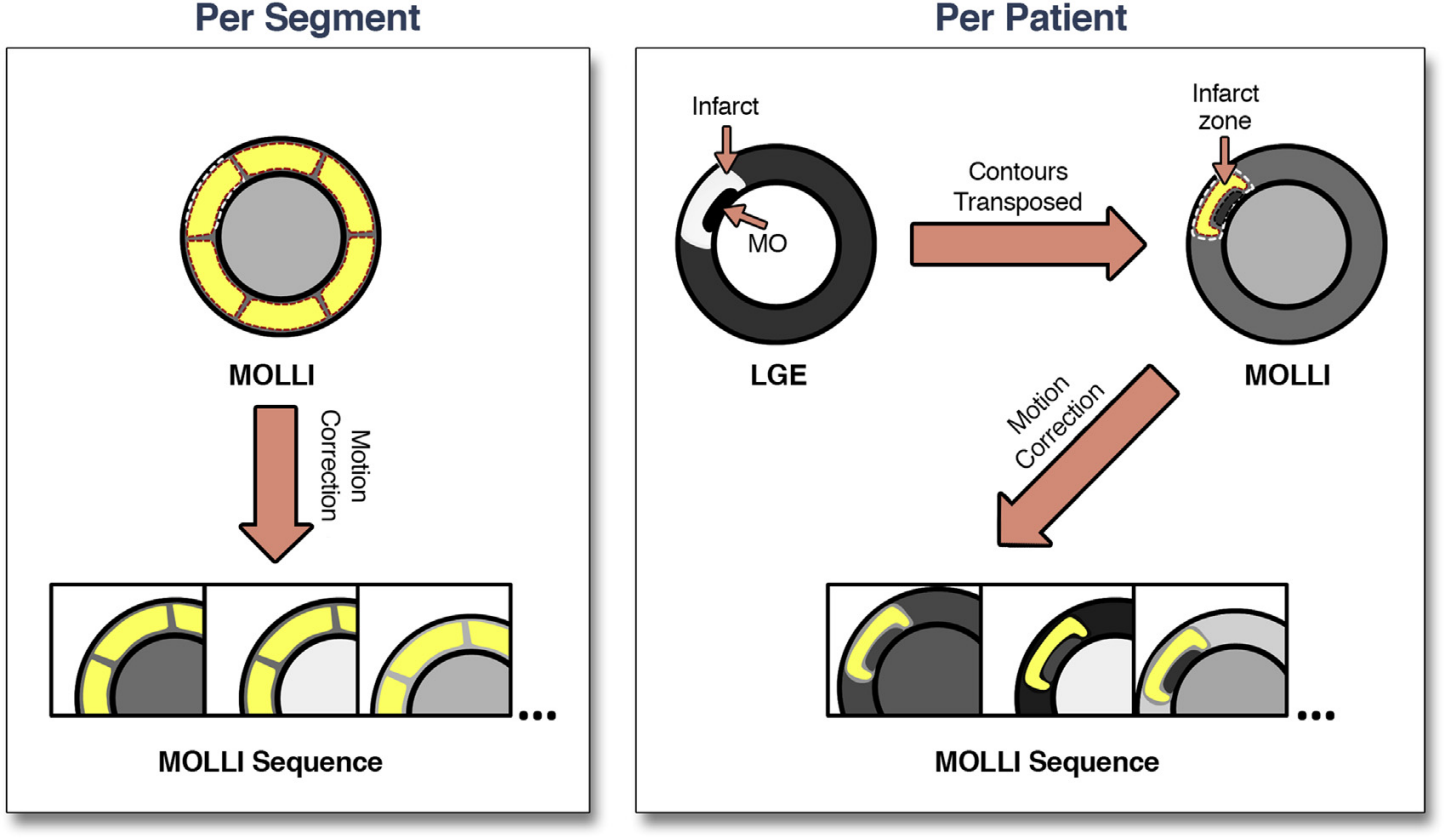
Estimation of Infarct ECV In the per-segment analysis **(left)**, ECV is taken in segments throughout the myocardium (16 segments in 3 short-axis slices, and these regions of interest are motion corrected in the MOLLI sequence before T1 estimation. Pre- and post-contrast T1 images are used to derive ECV. In the per-patient analysis **(right)**, the infarct zone is determined by signal intensity analysis on LGE images, which is then transposed to MOLLI images. Areas of MO are excluded, as are pixels at a tissue interface that may be susceptible to partial volume effects to highlight the infarct zone **(yellow contour)**. This contour is motion corrected for all images in the MOLLI sequence. ECV = extracellular volume; LGE = late gadolinium enhancement; MO = microvascular obstruction; MOLLI = modified Look-Locker inversion.

**Figure 2:**
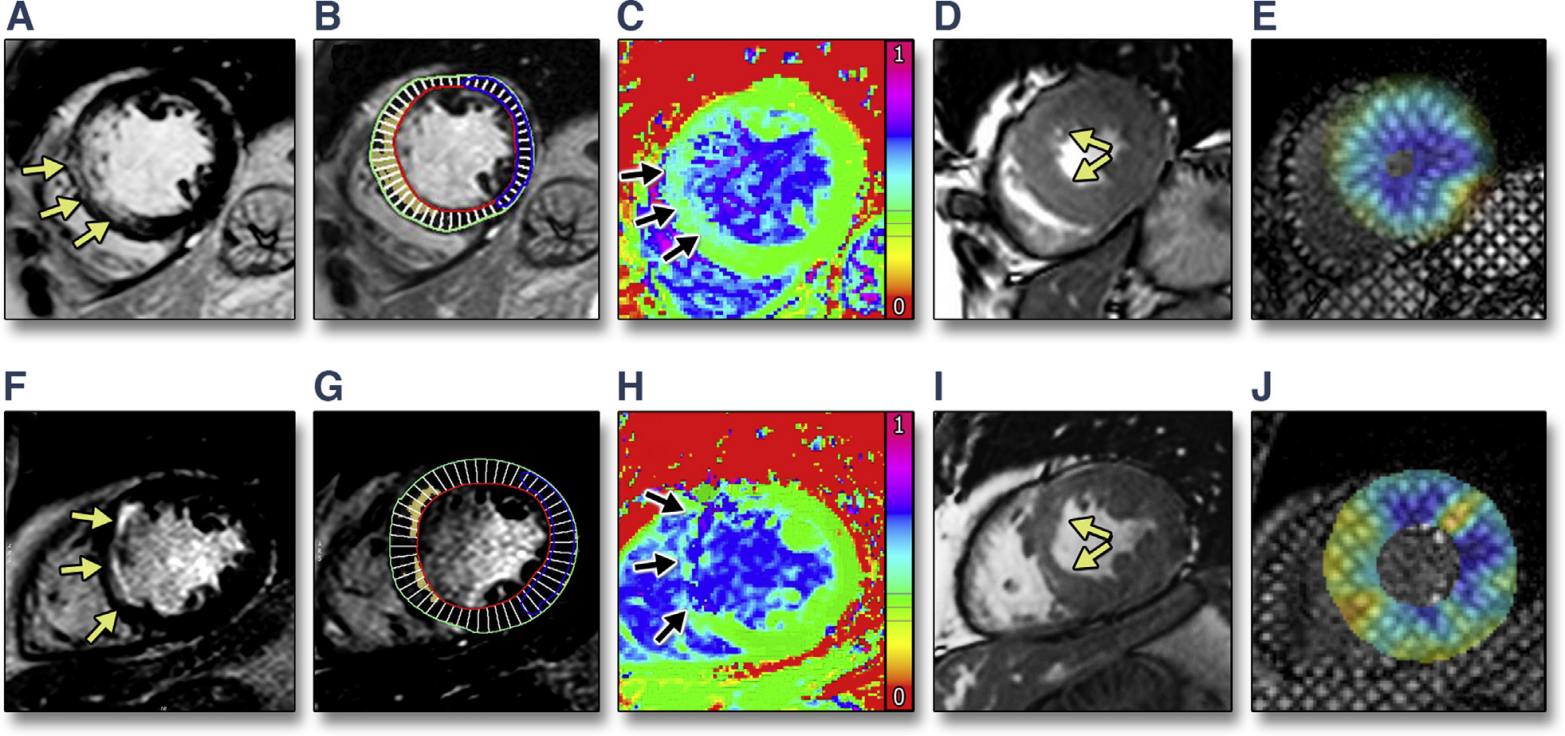
Comparison of LGE Transmural Extent and ECV With Regional Function and Strain **(Top)** A patient with high acute infarct transmural extent (78%, **arrows**) on LGE **(A)** as quantified by threshold analysis **(B)**. There is relatively low infarct ECV (0.44), as illustrated here by the modest gradation from normal myocardial ECV **(yellow/green, arrows)** on an ECV map **(C)**. Recovery of function is good at 3 months, as seen on cine imaging (systolic image, **D, arrows**) and normal peak systolic strain (blue myocardium, **E**). **(Bottom)** A patient with modest transmural extent of infarction acutely **(F and G)** has high acute ECV within the infarct zone (0.70, **H, arrows**). A septal regional wall motion abnormality persists at 3 months (systolic cine image, **I, arrows**) with decreased strain septally (yellow myocardium, **J**). Abbreviations as in Figure 1.

Images were analyzed offline using commercial software (cvi42 version 4.1.3, Circle Cardiovascular Imaging, Calgary, Canada; and inTag version 1.0, CREATIS lab, Lyon, France). Two types of analysis were performed. To evaluate performance of ECV estimation independent of LGE imaging, segmental analysis was performed using 3 short-axis slices and by analyzing each segment of the modified 16-segment American Heart Association model (12) (Figure 1). To evaluate the relative performance of ECV in conjunction with LGE imaging to reflect the clinical setting, a per-patient analysis was also performed using 1 region of interest each for infarct and remote zones per patient (Figure 1). The short-axis slice with the largest infarct size per patient on the acute visit was selected, with the entire infarct zone selected on this slice. The same slice was used in the convalescent scan. Patients with maximal scar extent <2 × 2 voxels of the in-plane resolution of LGE and T1 mapping were deemed too small for accurate evaluation of the infarct zone and not included in the per-patient analysis (but were included in segmental analysis). For both analyses, transmural extent of infarction was quantified to the nearest 5% using a modified centerline method with 100 chords within each short-axis LGE slice (13) (Figure 2).

LGE signal intensity was recorded in arbitrary units as generated by cvi42 analysis software (Online Appendix).

Regional wall motion anomaly was graded per-segment and per-patient from cine imaging by an experienced cardiologist (A.K., 4 years’ CMR experience), blinded to the results of strain and LGE and scored as: 0 = normal; 1 = mild or moderate hypokinesis; 2 = severe hypokinesis; 3 = akinesis; 4 = dyskinesis (3).

Myocardial ECV was calculated from native and post-contrast MOLLI images (14). For the per-segment analysis, each of 16 segments had mean ECV evaluated, blinded to the results of LGE and taking care to avoid partial-volume interaction with blood pool or pericardium. Regions of interest were manually motion corrected as required. For the per-patient analysis, T1 was calculated for infarcted and remote myocardium using a region of interest within the infarct and remote zone, with a conservative region of interest, and avoiding partial-volume effects from neighboring tissue, MO, or blood pool (Figure 1). The fit of the T1 curve was assessed; for both methods, regions with R^2^ < 0.95 were rejected.

Mid-myocardial end-systolic circumferential strain was measured through the infarct and remote zones using tissue tagged imaging (Figure 2).

Detailed image analysis techniques are described in the Online Appendix.

### Statistical analysis

Statistical analysis was performed using IBM SPSS Statistics 21.0 (Armonk, New York). Continuous variables are expressed as mean ± SD, and compared using paired Student *t* tests. All tests were 2-tailed; p values <0.05 were considered significant. Qualitative measures were correlated using the Spearman rank test; quantitative measures were correlated with the Pearson coefficient. Regression and power calculation details are outlined in the Online Appendix.

## Results

### Patient characteristics

Recruitment details are given in the Online Appendix. Thirty-nine patients completed baseline and follow up scans, and were included in the statistical analysis. Patient demographics are shown in Table 1. No sex-based differences were present. Four patients had scar volume too small for accurate infarct ECV quantification in the per-patient analysis; data from these patients were included in the per-segment analysis.

**Table 1:** Patient Characteristics (N = 39)

Age, yrs	57 ± 11
Male	34 (87)
Body mass index, kg/m^2^	27.9 ± 3.20
Current smoker	26 (67)
Hypertension	8 (21)
Hypercholesterolemia	10 (26)
Diabetes mellitus	4 (10)
Pain to balloon time, min	221 [275]
Infarct territory	
Anterior	19
Inferior	17
Lateral	3
Microvascular obstruction	17 (44)
TIMI flow grade ≥2 pre-PCI	3 (9)
TIMI flow grade 3 post-PCI	39 (100)
Peak troponin I, ng/l, median	>50,000
Peak CK, IU/l	905 [1,767]
Baseline CMR scan, days	2 [1]
Follow up CMR scan, days	102 [19]

Values are mean ± SD, n (%), or median [interquartile range].

CK = creatine kinase; CMR = cardiac magnetic resonance; PCI = percutaneous coronary intervention; TIMI = Thrombolysis In Myocardial Infarction.

### Infarct characteristics

Infarct characteristics are shown in Table 2. Infarct native T1 significantly decreased with time (Table 2), and was significantly higher in infarct than remote myocardium (p < 0.001 at day 2 and at 3 months). The infarct zone acutely demonstrated a wide ECV range between patients (range 0.34 to 0.85). Acute infarct ECV in patients with and without MO (per-patient ECV measurement excluded any MO zone) was similar (0.54 ± 0.18 vs. 0.57 ± 0.10; p = 0.6), acute LGE transmural extent was nonsignificantly raised in patients with MO (42 ± 17% vs. 33 ± 15%; p = 0.16). Acute infarct ECV and acute transmural extent of LGE did not correlate significantly (r = 0.34; p = 0.07) (Online Appendix, Online Figure 1). Transmural extent of LGE decreased significantly between acute and convalescent visits (Table 2).

**Table 2:** Infarct Characteristics

	Acute Visit	Convalescent Visit	p Value
Ejection fraction, %	49 ± 9	59 ± 7	<0.01
LV EDVi, ml/m^2^	81 ± 15	84 ± 20	NS
LV ESVi, ml/m^2^	41 ± 12	35 ± 13	<0.01
LV mass, g	129 ± 28	111 ± 28	<0.01
LGE transmural extent, %	33 ± 16	26 ± 11	<0.01
LGE infarct volume, ml	16 ± 11	10 ± 8	<0.01
LGE MO volume, ml	2 ± 2	—	—
Area at risk, ml	34 ± 14	—	—
Myocardial salvage index	0.56 ± 0.25	—	—
Infarct native T1	1,333 ± 110	1,244 ± 124	<0.01
Remote native T1	1,189 ± 75	1,146 ± 102	0.05
Infarct ECV	0.56 ± 0.14	—	—
Remote ECV	0.29 ± 0.06	—	—

Values are mean ± SD. Values given are from a per-patient analysis. Normal values for T1 and ECV are 1,052 ± 23 and 0.26 ± 0.04, respectively.

ECV = extracellular volume; EDVi = end-diastolic volume, indexed to body surface area; ESVi = end-systolic volume, indexed to body surface area; LGE = late gadolinium enhancement; LV = left ventricular; MO = microvascular obstruction.

### Per segment

**Figure 3:**
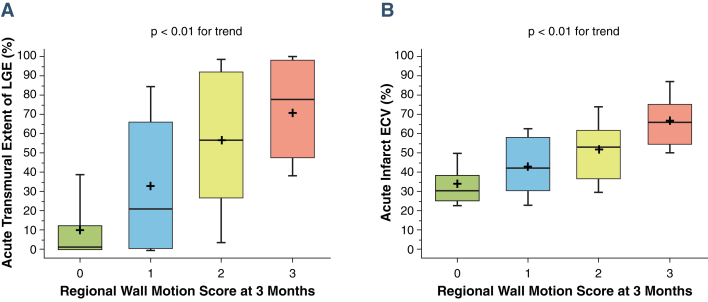
Comparison of Per-Segment Convalescent Regional Wall Motion Score With LGE Transmural Extent and Infarct ECV **(A)** LGE transmural extent; **(B)** infarct ECV. Note relatively wide ranges for transmural extent. **Box** denotes median and 25th and 75th percentiles; mean is indicated by a **plus sign**, and **whiskers** are at 9th and 91st percentiles.

**Figure 4:**
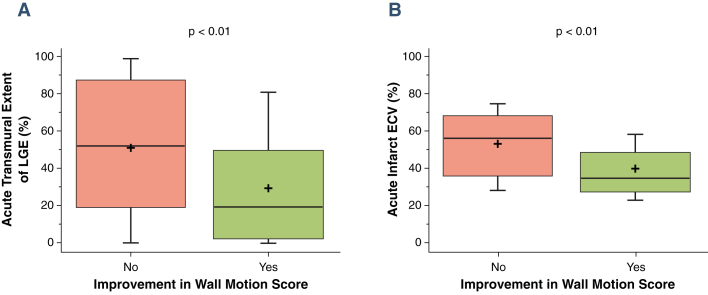
Acute Transmural Extent of LGE and Acute Infarct ECV Compared With Improvement in Wall Motion Score Over 3 Months **(A)** Acute transmural extent of LGE; **(B)** acute infarct ECV. Data for dysfunctional segments (n = 163) are shown. Abbreviations as in Figure 1.

**Figure 5:**
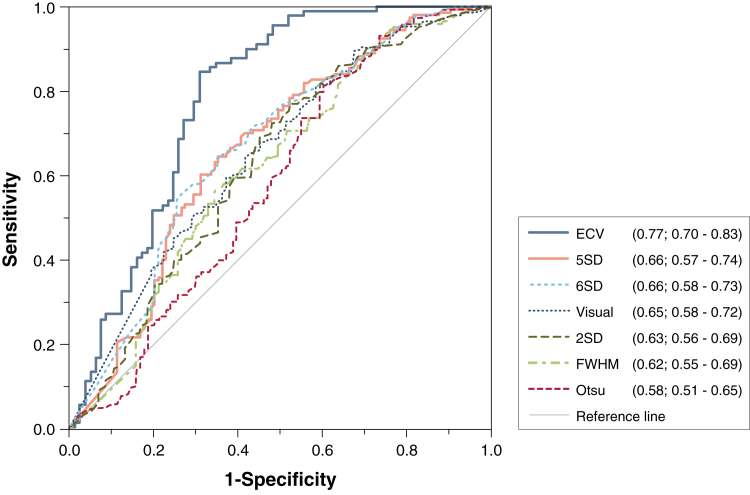
ROC Curve Comparing Infarct ECV and Transmural Extent of Infarction in Dysfunctional Segments (n = 163) With Improvement in Wall Motion Score at 90 Days Remote segments not shown. A variety of thresholds for LGE transmural extent are shown. C-statistic (AUC) and 95% confidence interval is shown in the legend for each method. AUC = area under the curve; FWHM = full-width half-maximum; ROC = receiver-operating characteristic; other abbreviations as in Figure 1.

**Figure 6:**
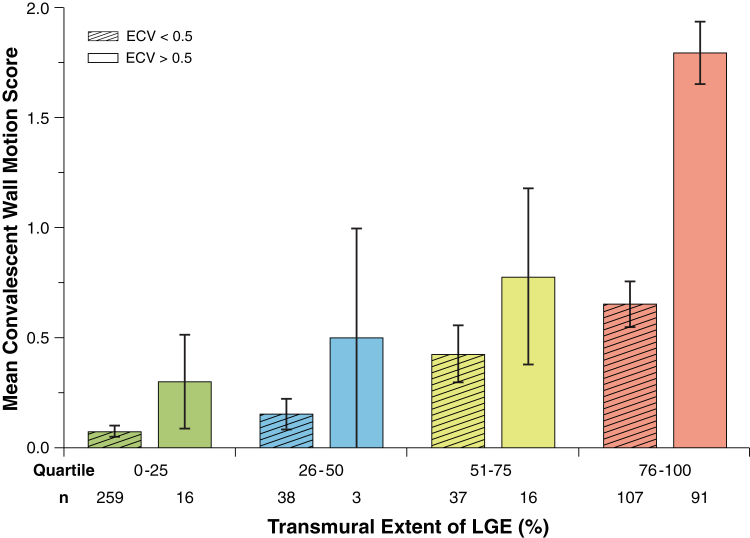
Segmental Analysis Comparing Each Quartile of Acute LGE Transmural Extent With Mean Convalescent Wall Motion Score Groups are split by a cutoff value of 0.5 for acute ECV. A higher score indicates more severe segmental dysfunction. The **left bar** in each quartile represents patients with ECV <0.5; the **right bar** represents ECV >0.5. Abbreviations as in Figure 1.

Acute infarct ECV correlated with wall motion score acutely (β = 0.47, r = 0.47; p < 0.01) and at 90 days (β = 0.55, r = 0.54; p < 0.01) (Figure 3). Improvement in wall motion score decreased with increasing ECV (F = 23.0; p < 0.01) (Figure 4). Acute transmural extent of LGE also correlated with wall motion score both acutely (β = 0.55, r = 0.55; p < 0.01) and at 90 days (β = 0.51, r = 0.50; p < 0.01) (Figure 3). In dysfunctional segments, improvement in wall motion score decreased with increasing acute transmural extent of LGE (F = 6.4; p < 0.01) (Figure 4). Receiver-operating characteristic curve analysis for the prediction of improvement in wall motion score at 90 days demonstrated a significantly higher area under the curve (AUC) for acute infarct ECV than acute transmural LGE extent (0.77 [95% confidence interval: 0.70 to 0.83] vs. 0.66 [95% confidence interval: 0.57 to 0.74]; p = 0.02) (Figure 5). ECV had significantly higher AUC than LGE for all thresholds measured (p < 0.05 for all) (Figure 5). Infarct ECV of ≤0.5 had sensitivity 81% and specificity 65% for prediction of improvement in segmental function. Adding acute ECV analysis to a 50% LGE transmural extent cutoff for prediction of wall motion improvement in dysfunctional segments increased sensitivity from 61% to 75% and specificity from 71% to 85%, and showed a trend toward improved prediction of convalescent wall motion score (Figure 6) and functional recovery (Online Figure 2) across the range of LGE transmural extent.

### Per patient

**Figure 7:**
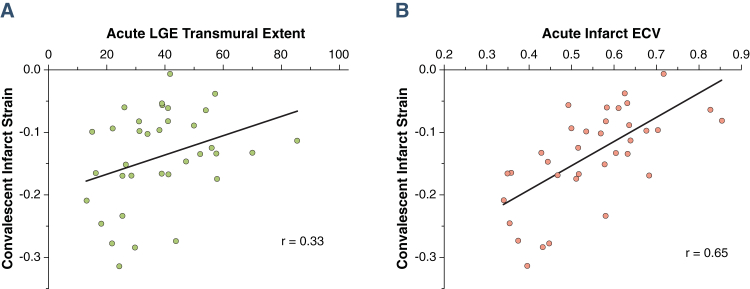
Comparison of Convalescent Infarct Zone Strain With Acute Transmural Extent of LGE and Acute Infarct ECV **(A)** Acute transmural extent of LGE; **(B)** acute infarct ECV. Per-patient data (n = 35) shown. Abbreviations as in Figure 1.

Performance of acute ECV and acute LGE to predict markers of LV function per patient are shown in Table 3. ECV and LGE correlated with convalescent wall motion score (r = 0.45; p < 0.01; r = 0.41; p = 0.02, respectively) and convalescent ejection fraction (EF) (r = −0.56; p < 0.01; r = −0.34; p = 0.04, respectively). Signal intensity of acute LGE did not correlate with convalescent EF (r = −0.19; p = 0.3) or convalescent infarct strain (r = 0.16; p = 0.4). ECV had numerically higher correlation than LGE transmural extent for all per-patient markers of convalescent LV function, including infarct zone strain (Figure 7, Table 3), though the comparisons of correlation coefficients did not reach statistical significance.

**Table 3:** Correlation Between Acute LGE Transmural Extent and Acute ECV per Patient With Markers of Acute and Convalescent LV Function

Marker of LV Function	Acute LGE		Acute ECV	
	Correlation Coefficient, r	p Value	Correlation Coefficient, r	p Value
Acute wall motion score	0.37	0.03	0.34	0.049
Convalescent wall motion score	0.41	0.02	0.45	<0.01
Acute infarct strain	0.24	0.15	0.27	0.11
Convalescent infarct strain	0.33	0.06	0.65	<0.01
Convalescent ejection fraction	−0.34	0.04	−0.56	<0.01

Abbreviations as in Table 2.

### Regression analysis

Univariable linear regression analysis (examining per-patient the variables in Table 4) showed that acute infarct ECV, but not acute LGE transmural extent, was significantly associated with attenuated convalescent infarct zone strain. In multivariable linear regression analysis, ECV was independently associated with EF (β = −0.33; p = 0.04), whereas transmural extent was not (β = −0.18; p = 0.24) (Table 4). Additionally, acute infarct ECV showed significant independent association with convalescent infarct zone strain (β = 0.38; p = 0.03), whereas acute transmural extent of LGE did not (β = −0.19; p = 0.29).

**Table 4:** Predictors of Decreased Infarct Zone Strain and EF in Univariable and Standard Multivariable Regression Analysis

	Convalescent Strain				Convalescent EF			
	Univariable		Multivariable		Univariable		Multivariable	
	R	p Value	β	p Value	R	p Value	β	p Value
Age	−0.02	0.92	—	—	−0.07	0.69	—	—
Sex	0.19	0.28	—	—	0.40	0.02	0.16	0.18
Current smoker	0.02	0.92	—	—	0.08	0.64	—	—
Hypertension	0.01	0.95	—	—	0.20	0.25	—	—
Hypercholesterolemia	0.10	0.56	—	—	0.03	0.87	—	—
Diabetes	0.31	0.09	0.20	0.12	0.06	0.74	—	—
Heart rate at CMR	0.01	0.94	—	—	−0.16	0.36	—	—
Blood pressure, systolic/diastolic	−0.03/0.05	0.9/0.8	—	—	−0.01/−0.09	0.9/0.6	—	—
Pain onset to balloon time	0.16	0.35	—	—	−0.09	0.18	—	—
Anterior AMI	−0.08	0.63	—	—	−0.14	0.42	—	—
TIMI flow grade before PCI	−0.31	0.07	0.11	0.43	0.17	0.32	—	—
TIMI flow grade after PCI	0.00	1.00	—	—	0.00	1.00	—	—
LV mass (indexed to BSA)	0.23	0.18	—	—	−0.30	0.08	−0.09	0.64
Remote mid-myocardial circumferential strain	0.24	0.16	—	—	0.01	0.94	—	—
Myocardial salvage index	−0.45	0.06	−0.24	0.15	0.39	0.02	0.10	0.40
Area at risk	−0.22	0.20	—	—	−0.02	0.91	—	—
Infarct LGE transmural extent	0.33	0.05	−0.19	0.29	−0.34	0.05	−0.18	0.24
Infarct ECV	0.65	<0.0005	0.38	0.03	−0.56	0.001	−0.33	0.04

Variables are taken from acute visit. Multivariable standardized regression coefficient (β) and p values are shown where the variable was included in the multivariable analysis.

AMI = acute myocardial infarction; BSA = body surface area; other abbreviations as in Tables 1 and 2.

## Discussion

This study shows that the ECV in acute reperfused myocardial infarction is predictive of regional and global LV functional recovery, and adds prognostic value to LGE. ECV adds quantitative information about severity of myocardial injury within the infarct zone to the assessment of infarct extent by LGE. Particularly for infarcts with higher transmural extent, acute infarct ECV appears to be an additional predictor of functional recovery that can complement transmural infarct extent by LGE.

ECV provides a continuous measure of tissue composition and thus a potential tool to interrogate the severity of myocardial damage in infarcted myocardium. Acute ECV was a stronger predictor than LGE for convalescent EF and attenuated strain in the infarct zone; when correcting for ECV, LGE was no longer an independent predictor of EF or infarct zone strain. ECV demonstrated utility throughout the range of infarct severity observed in reperfused AMI. ECV-derived measurement of infarct severity also complemented LGE measurements of infarct extent, and increased the sensitivity and specificity to predict functional improvement in patients with >50% transmural LGE that was independent of LGE analysis. ECV had higher accuracy than LGE to predict improvement in regional wall motion score (Figure 5), higher degree of correlation with infarct zone strain (Figure 6), and reduced spread of values across the range of wall motion abnormalities (Figure 3).

LGE is established as a clinical reference standard for viability imaging following AMI, but accuracy may be reduced when using the technique in this context. Recent European guidelines note that prediction of functional recovery with LGE CMR is “no better than other imaging techniques” (15). Choi et al. (16) compared acute transmural extent of LGE with convalescent function in 24 patients following reperfused ST-segment elevation myocardial infarction. Their observation of decreasing functional recovery with increasing transmural extent was driven largely by segments with either 0% or 100% transmural extent, which made up approximately two-thirds of the sample. Ingkanisorn et al. (6) found a significant reduction in wall thickening with increasing transmural extent, but no significant difference in the quartiles between 1% to 75% transmurality. A comparable analysis in 30 patients demonstrated that 25% of segments with 75% to 100% acute transmural infarction had functional improvement at 13 weeks (17). Gerber et al. (7) observed good correlation between infarct transmural extent and segmental strain measurements. Finally, Shapiro et al. (18) found that transmural extent of LGE was predictive of functional outcome in 17 patients, even accounting for presence of MO. Results of the present study were consistent with these previous reports and showed that higher transmural extent of scar was associated with impaired wall motion both acutely and at 90 days (Figure 3) and with lower improvement in wall motion score over time (Figure 4). However, these previous reports and our data also indicate that, whereas LGE can accurately predict functional outcome in AMI in areas of no LGE or with full transmural infarction, its accuracy is reduced in intermediate (25% to 75%) transmural infarct extent. Peri-infarct edema and remodeling of the infarct zone over time may lead to comparatively high transmural extent acutely 4, 16, 19, 20. Additionally, LGE cannot differentiate degrees of severity of tissue damage within the hyperenhanced infarct zone. By contrast, this study uses ECV, not to delineate spatial extent of infarction, but as a measure of infarct severity. Our data suggest that ECV can provide characterization in the diagnostic quandary of intermediate LGE extent, and adds an additional dimension to assessment of infarct transmurality by LGE. In histological studies, myocardial infarcts maintain foci of preserved myocytes within areas of necrosis (20), raising potential for functional recovery. Interstitial expansion in infarction is also variable and may depend on the extent of local reperfusion (19). These observations may, in part, explain contractile recovery found within the infarct zone 7, 8, and the range of infarct ECV values observed in the present study.

ECV estimation by CMR is validated in models of chronic fibrosis rather than acute infarction (14). Studies to date have mainly focused on native T1 in acute infarction, with similar findings to this study. Messroghli et al. (21) found that native T1 maps were sensitive to acute infarction, but did not correlate this with functional recovery. Dall’Armellina et al. (22) found that native T1 was correlated with functional recovery on a segmental basis, and accuracy of T1 was not limited by intermediate values in the same way as transmural extent of LGE. ECV has been measured in chronic infarction, and ECV maps have previously been demonstrated to delineate areas of infarction 9, 23. For both LGE and ECV, the pathophysiological correlates in AMI are less well established, but both methods are likely to detect the expanded interstitial space within the infarct zone arising from cell death with or without edema or intracellular contrast uptake (24).

Despite the clear association of ECV and functional recovery, several potential confounders should be considered. Edema affects both T1 and ECV (25) and is an important determinant of recovery in the peri-infarct zone (26) and also occurs within infarcted myocardium (20), but differentiating edema from scar within the core of the infarct zone cannot be accurately performed with current CMR techniques. Our findings are in keeping with previous observations that peri-infarct edema and myocardial salvage are associated with improved regional and global LV function (26); however, ECV, but not LGE, remained a significant predictor even when accounting for peri-infarct edema (Table 4). It is known that MO and intramyocardial hemorrhage are independent markers of poor functional outcome 7, 8. In the present study, the per-patient analysis deliberately excluded areas of visible MO from ECV measurements, because contrast equilibrium (a prerequisite for ECV estimation by T1) is not achieved in these areas. Care was taken to measure ECV at least 1 voxel away from noninfarct tissue to minimize partial volume errors (Figure 1). We defined infarct size separately for baseline and follow-up visits rather than transposing contours from convalescence to acute studies (26). This approach allowed us to test the clinically relevant predictive value of acute LGE, which a retrospective transposition would not have allowed.

### Study limitations

The sample size in this study is relatively small, but in keeping with similar studies in this demographic 6, 7, 16 and adequately powered to detect the significant differences observed. Fully adjusting for demographic and other infarct variables would require larger, likely multicenter studies, but is less relevant when comparing 2 simultaneously acquired imaging markers in the same patients. This study was not specifically powered to evaluate the interaction between LGE and ECV measurements. Four patients with minimal scar following reperfusion were deemed unsuitable for per-patient analysis; all of these patients had normal wall motion acutely and in convalescence. Equilibrium-contrast ECV estimation may be more accurate for high ECV values than a bolus method (14); however, the bolus method used in this study can be more easily integrated into existing clinical protocols. The optimal threshold for hyperenhancement is debated 27, 28; in the present study, we therefore deliberately evaluated the optimal threshold given our setup and pulse sequence.

## Conclusions

This study demonstrates that addition of CMR-derived ECV estimation of the infarct zone after AMI offers increased accuracy to predict ejection fraction and functional recovery compared with LGE alone. Acute ECV estimation is feasible in this demographic and can provide clinically relevant information. The potential clinical utility of infarct ECV mapping post-AMI is most pronounced in cases of intermediate LGE transmural extent, and potentially allows for improved early characterization and prognostication of patients post-AMI.

Perspectives **COMPETENCY IN MEDICAL KNOWLEDGE:** CMR can be used to predict the amount of contractile recovery after reperfused AMI. The transmural extent of scar as measured from LGE CMR is a well-established predictive marker of functional recovery. The novel quantitative measure of myocardial ECV is compared with LGE for the ability to predict contractile recovery in AMI. Both LGE and ECV at day 2 were predictive of contractile recovery at 3 months. ECV had higher accuracy than LGE to predict improved convalescent wall motion. An acute infarct ECV of >0.5 suggested poor contractile recovery; ECV <0.5 predicted contractile recovery with sensitivity 81% and specificity 65%. After accounting for interactions, ECV was independently associated with measures of convalescent contractility; LGE was not. Measurement of ECV by CMR is a useful additional tool in the setting of reperfused AMI, to help predict the degree of contractile recovery.

**TRANSLATIONAL OUTLOOK:** Further multicenter studies with different CMR vendors are warranted to determine the optimal method to combine the two measures of LGE and ECV. Future work should characterize the pathophysiological correlates of ECV variability in AMI.
