# Supplementary material for: Myocardial Extracellular Volume Estimation by CMR Predicts Functional Recovery Following Acute MI
Source: JACC Cardiovasc Imaging. 2017 Sep;10(9):989–99. doi: 10.1016/j.jcmg.2016.06.015 (PMC5593809; doi:10.1016/j.jcmg.2016.06.015)
Supplement: Online Data [file mmc1.docx]

**ONLINE APPENDIX**

**Supplementary Methods**

Pulse sequence parameters and CMR protocol

Cine imaging used a balanced steady-state free precession (bSSFP) pulse sequence (echo time (TE)/repetition time (TR)/flip angle 1.3ms/2.6ms/40°, spatial resolution 1.6×2.0×10mm, typical temporal resolution 25ms). Tissue tagging used a spatial modulation of magnetization (SPAMM) pulse sequence (spatial resolution 1.5×1.5×10mm, tag separation 7mm, temporal resolution ≤35ms, typical TR/TE/flip angle 5.9/3.5/10°). T2-weighted imaging used a dark-blood T2w fast spin echo short tau inversion-recovery (STIR) sequence (TE 90 ms, TR two R-R intervals, flip angle 90 degrees, spatial resolution 1.7×1.7×10 mm) and constant level appearance (CLEAR) homogeneity correction. T1 mapping used a 17-heart MOLLI method to acquire 11 images (3-3-5 acquisition with 3x R-R interval recovery epochs) in a single end-expiratory breath hold (1,2) (bSSFP readout in end-diastole, manual RF shim adjustments, 2x sensitivity-encoded (SENSE) acceleration, acquired spatial resolution 1.7×2.1×10mm, typical TE/TR/flip angle 2.7/1.06/35°). 0.1mmol/kg gadolinium-DTPA (gadopentetate dimeglumine; Magnevist, Bayer, Berlin, Germany) was then administered using a power injector (Spectris, Solaris, PA). Repeat MOLLI T1 mapping was performed at 15 minutes post-contrast, and LGE imaging at 16-20 minutes post contrast (inversion recovery-prepared T1-weighted gradient echo, inversion time according to Look-Locker scout, spatial resolution 1.54×1.75×10mm, TR/TE/flip angle 3.7/2.0/25°). In addition to the three LGE short-axis slices for analysis, a contiguous stack of LGE images was acquired immediately after the three slice LGE acquisition (imaging parameters as above, slice thickness 10mm, no slice gap).

## Image analysis

In addition to alignment of slices during image acquisition, accurate alignment of serial scans was verified by comparing ventricular and papillary muscle morphology. Slices that were not aligned between sequences or time-points were not analyzed. Left ventricular volumes and ejection fraction (EF) were analyzed from cine images using standard methods. Infarct location was determined by CMR, according to standard guidelines (3). MO was defined visually as the hypointense core within the infarct zone on LGE imaging. A variety of LGE thresholding techniques (including any hypointense core) for identification of infarcted myocardium were compared for segmental analysis, namely 2, 5 and 6 standard deviations (SD), visual infarct delineation (blinded to the results of other threshold techniques), full-width at half maximum (FWHM) and a histogram-based automated threshold technique (Otsu) (4). Infarcted myocardium defined as LGE hyperenhancement ≥5 standard deviations (SD) above remote myocardium and including any hypointense core was chosen for further comparisons as it had the highest c-statistic for prediction of recovery of segmental function. Infarct size and MO were measured from LGE images. Infarct volumes were calculated across the whole LV stack by the modified Simpson’s method.

LGE transmural extent chord data were averaged into either 16-segments for the segmental analysis, or averaged across the infarct zone for per-patient analysis. Segmental analysis for improvement in function included only those segments that were dysfunctional (wall motion score ≥1) acutely. Remote myocardium was selected as a 60° sector diametrically opposing the infarct and in an area without LGE. LGE signal intensity was analyzed from the scanner-generated DICOM images, with the signal intensity obtained as a mean of the infarct zone using the same region of interest as in the per-patient analysis, and recorded in arbitrary units as generated by cvi42 analysis software.

ECV was calculated for infarct and remote zones using the following equation, where R1 is the reciprocal of T1 (5):

$$ECV= \left( 1-hematocrit \right)\times\frac{{R1}_{myo post}-{R1}_{myo pre}}{{R1}_{blood post}-{R1}_{blood pre}}$$

T1 and ECV normal reference ranges for our center/sequence have been published previously (6).

Strain was measured in the single short-axis slice that demonstrated maximal infarction on LGE imaging. The same position was analyzed for the convalescent scan. Area at risk was measured from T2-weighted imaging, with delineation of edematous myocardium using the full-width at half maximum threshold technique. Myocardial salvage index was calculated using the formula: myocardial salvage index = (area at risk – infarct volume) / area at risk.

Statistical analysis

Differences in segmental measurements were evaluated using a multilevel linear mixed-effects model to account for non-independence of segmental data; r denotes partial correlation in this context. A sample size of 35 patients was calculated (7) to detect a 10% difference in accuracy (AUC) with power 80% at a significance level of 0.05, assuming rank correlation of 0.55 between ECV and transmural extent of LGE, accuracy of LGE as 0.82, artefact in 15% of segments, and one-third of segments dysfunctional acutely (8). Normality for quantitative data was established using the Kolmogorov-Smirnov test. Univariable analyses were performed to identify predictors of reduced strain and EF at 90 days. Variables with a probability value <0.1 in the univariable analysis were included in a multivariable linear regression analysis. Receiver operating characteristic (ROC) curves were compared using the method described by DeLong et al. (9) The optimal cut point for ECV imaging was determined from the ROC curve (Figure 5) using Youden’s index (10).

**Supplementary Results**

Patient Recruitment

Of 90 eligible patients admitted within the recruitment period, 13 had obesity that precluded CMR, 4 were unable to lie flat, 1 had an MR-incompatible pacemaker, 14 were unable to consent and 5 were due CABG prior to the follow-up scan. The remaining 53 patients were approached, of which 9 declined to participate. Therefore 44 patients constituted the study sample. 5 did not attend follow-up. 39 patients therefore completed baseline and follow up scans and were included in the statistical analysis. 18 slices (15%) did not have good alignment between methods and/or time-points and were excluded from analysis. 7 segments in segmental analysis (all remote) were excluded as R^2^ for T1 mapping was <0.95. All regions in the per-patient analysis had R^2^≥0.95.

**Supplementary References**

1. Messroghli DR, Radjenovic A, Kozerke S, Higgins DM, Sivananthan MU, Ridgway JP. Modified Look-Locker inversion recovery (MOLLI) for high-resolution T1 mapping of the heart. Magn Reson Med 2004;52:141-6.

2. Messroghli DR, Walters K, Plein S et al. Myocardial T1 mapping: application to patients with acute and chronic myocardial infarction. Magn Reson Med 2007;58:34-40.

3. Cerqueira MD, Weissman NJ, Dilsizian V et al. Standardized myocardial segmentation and nomenclature for tomographic imaging of the heart. A statement for healthcare professionals from the Cardiac Imaging Committee of the Council on Clinical Cardiology of the American Heart Association. Circulation 2002;105:539-42.

4. Otsu N. A Threshold Selection Method from Gray-Level Histograms. Systems, Man and Cybernetics, IEEE Transactions on 1979;9:62-66.

5. Miller CA, Naish JH, Bishop P et al. Comprehensive validation of cardiovascular magnetic resonance techniques for the assessment of myocardial extracellular volume. Circ Cardiovasc Imaging 2013;6:373-83.

6. Dabir D, Child N, Kalra A et al. Reference values for healthy human myocardium using a T1 mapping methodology: results from the International T1 Multicenter cardiovascular magnetic resonance study. J Cardiovasc Magn Reson 2014;16:69.

7. Hanley JA, McNeil BJ. A method of comparing the areas under receiver operating characteristic curves derived from the same cases. Radiology 1983;148:839-43.

8. Romero J, Kahan J, Kelesidis I et al. CMR imaging for the evaluation of myocardial stunning after acute myocardial infarction: a meta-analysis of prospective trials. Eur Heart J Cardiovasc Imaging 2013;14:1080-91.

9. DeLong ER, DeLong DM, Clarke-Pearson DL. Comparing the areas under two or more correlated receiver operating characteristic curves: a nonparametric approach. Biometrics 1988;44:837-45.

10. Youden WJ. Index for rating diagnostic tests. Cancer 1950;3:32-5.

**Supplementary Figures**

**Figure 1.** Comparison of ECV with LGE transmural extent by quartiles. Data presented are per-segment.

**Figure 2.** Prediction of improvement in convalescent wall motion score for each quartile of acute LGE transmural extent, using a cut-off value of 0.5 for acute ECV. Segmental analysis of dysfunctional segments only (n=163). The first bar in each quartile represents patients with ECV <0.5; the second bar represents ECV >0.5.
